# Recruitment and Enrollment of a National Sample of Transgender Youth via Social Media: Experiences from Project Moxie

**DOI:** 10.1089/trgh.2018.0062

**Published:** 2019-07-25

**Authors:** Michael Miller-Perusse, Keith J. Horvath, Tanaka Chavanduka, Rob Stephenson

**Affiliations:** ^1^ Center for Sexuality and Health Disparities, School of Nursing, University of Michigan, Ann Arbor, Michigan.; ^2^ Division of Epidemiology and Community Health, School of Public Health, University of Minnesota, Minneapolis, Minnesota.; ^3^ Department of Systems, Population and Leadership, School of Nursing, University of Michigan, Ann Arbor, Michigan.

**Keywords:** transgender, youth, HIV, online recruitment, social media

## Abstract

This report compares social media strategies for enrolling transgender youth (TY) into online HIV prevention research. Over 12 months, 202 TY enrolled in Project Moxie, a randomized trial of an at-home HIV testing intervention. Free Craigslist advertisements showed promising success in enrolling TY, especially those of color. Paid Facebook advertising was successful in reaching a large sample of TY, as was participant referral. This supports previous literature suggesting peer referral as an effective strategy for reaching TY. High levels of attempted fraud were detected and mitigated. Findings demonstrate that recruitment and enrollment of a diverse TY sample is possible online.

## Introduction

Transgender youth (TY; 15–24 years) face a multitude of health disparities that increase their vulnerability to poor health outcomes.^1,2^ Although sexual and gender minority (SGM) populations are often regarded as “hidden,” online recruitment has been lauded as a tool for successfully engaging hard-to-reach populations in sexual health research.^3–6^ Online interventions have gained popularity in HIV prevention research, specifically with regard to SGM youth, yet their success depends on the ability to recruit and enroll diverse samples while detecting fraudulent activity.^5^ Recent reports show 95% of teens aged 13–17 years and 88% of adults aged 18–29 years use some form of social media, indicating a wide potential reach of online recruitment.^7,8^

Previous findings suggest that SGM youth spend more time online than do their non-SGM counterparts.^9^ Furthermore, researchers have observed a significant role of social media in identity formation and community building for SGM individuals, and specifically TY.^10–14^ Collectively, this suggests that online recruitment through social media may be a successful method to reach TY given its appeal and cultural relevance. Despite the widespread use of social media recruitment, little is known about the feasibility and success of recruiting TY across different platforms. This report compares enrollment rates and sample characteristics between online strategies for recruiting TY in an HIV prevention study (Project Moxie), with the aim of highlighting relative merits for several popular social media platforms.

## Methods

Project Moxie is a randomized trial that aims to test the feasibility of pairing a HIPAA secure, online video counseling intervention with at-home HIV testing for TY. A comprehensive study protocol was approved by the University of Michigan Institutional Review Board (IRB; HUM00123412) and is available elsewhere.^15^

After recruitment, participants were asked to take a baseline survey before randomization into two study arms: (1) the control arm, in which participants were sent an OraQuick at-home rapid HIV test and asked to report their results in an online study portal, or (2) the intervention arm, in which participants were sent an OraQuick at-home rapid HIV test and asked to participate in a remote motivational interviewing/counseling, testing, and referral (MI/CTR) session. MI/CTR sessions were completed using VSee, an online HIPAA secure video calling service. The study protocol was approved by the University of Michigan Institutional Review Board (IRB; HUM00123412) and is available elsewhere.^15^

Recruitment of TY took place from June 2017 to June 2018, using advertisements and postings placed on the following social media websites: Facebook, Instagram, Twitter, Tumblr, and Craigslist. Recruitment advertisements featured photos representing a spectrum of transgender and gender variant persons, and directing interested individuals to the Project Moxie website to earn up to $150 for participating.

The Project Moxie landing page provided basic study information, including a short description of activities, and an informed consent to screen for eligibility. Those who provided consent were directed to an eligibility screener. Eligible individuals then underwent a comprehensive online consent before participation. A waiver of need for parental consent to screen and enroll those under the age of 18 years was approved by the IRB.

Eligibility for the study included the following: (1) self-identification as noncisgender, indicated by a current gender identity differing from sex assigned at birth; (2) aged 15–24 years; (3) negative or unknown HIV status; (4) current U.S. residency; (5) willingness to receive an at-home HIV test; and (6) access to a computer, smartphone, or tablet that supports VSee video-calling software to implement the MI/CTR intervention. Pronouns and recruitment route were also asked at this time.

Eligible participants were instructed to create an account. All identifying information was stored on a password-protected server accessible only to IRB-approved study staff. Duplicate accounts and accounts that could be linked across inconsistent IP address, name, physical address, email, or phone number were flagged for verification. The information from these accounts was verified by staff using Spokeo, an online information aggregator; if the search was inconclusive, participants were contacted and asked to confirm their account information. After verification, participants were given the opportunity to refer friends into the study.

If multiple accounts were detected for a verified individual, they were notified by staff that their longest standing account would be retained while any others would be deleted. In the case of fraudulent activity—the persistent creation of numerous accounts with inconsistent information—the associated IP address was restricted from accessing the study server.

The current analysis describes the process of recruitment, rates of enrollment, demographic differences of participants, and associated costs across the social media and participant referral platforms. Proportion tests and Fisher's exact tests were conducted using Stata/SE 15.1 to determine statistically significant (*p* < 0.05) differences in enrollment rates and demographic data across recruitment platforms.

## Results

Project Moxie social media advertisements generated 1,113,955 impressions—the total number of times the advertisements were displayed to any user—resulting in 33,182 (3.0%) clicks. Electronic consent to screen for eligibility was obtained from a sample of 2707 individuals, 1365 (50.4%) of whom started the eligibility screener. Of these individuals, 698 (51.1%) met the study eligibility criteria. Reasons for ineligibility included self-identification as cisgender (275, 20.1%), age (303, 22.2%), HIV status (12, 0.9%), unwillingness to receive an at-home rapid HIV test (61, 4.5%), and lack of access to a computer (7, 0.5%).

Of the 698 eligible individuals, 480 (68.8%) consented for participation and created an account. Information from 216 (45%) accounts was verified, whereas 264 (55%) accounts were determined duplicate or fraudulent and dropped from the study. Of the 216 verified individuals who created accounts, 202 (93.5%) took the baseline survey through the study website.

For the purpose of this analysis, participants are categorized as enrolled once they have provided consent to screen for eligibility, met eligibility criteria, provided consent to participate, and taken the baseline survey. The largest volume of eligible (219, 31.4%) and enrolled (69, 34.2%) individuals came from Facebook, followed by participant referral (170, 24.3%; 57, 28.2%; Table 1). The overall sample had an enrollment rate of 28.9%, with a significantly greater proportion of eligible individuals enrolling when recruited through Craigslist (49.3%; *p*=0.000) and a significantly lesser proportion enrolling when recruited through Tumblr (7.3%; *p*=0.000).

**Table 1.** Eligibility and Enrollment by Platform

Platform	Screened eligible	Individuals enrolled	Enrollment rate^a^	Proportion test
	*n*=698	*n*=202	28.9	H_0_=28.9%
	% (*n*)	% (*n*)	%	*p*
Facebook	31.4 (219)	34.2 (69)	31.5	0.395
Instagram	9.7 (68)	10.9 (22)	32.4	0.584
Craigslist	10.2 (71)	17.3 (35)	49.3	**0.000**
Referral	24.3 (170)	28.2 (57)	33.5	0.183
Twitter	1.9 (13)	2.5 (5)	38.5	0.450
Tumblr	17.8 (124)	4.4 (9)	7.3	**0.000**
Local^b^	2.4 (17)	2.5 (5)	29.4	0.836
Missing	2.3 (16)	—	—	—

Bold indicates statistically significant values (*p*<0.05).

^a^ Percentage of those screened eligible who were successfully enrolled in the study.

^b^ Local sources of recruitment include on-site by study staff, newsletters/listservs, and palm card.

The Moxie participant sample was composed of mostly nonbinary individuals (83, 41.1%) and transgender men (82, 40.6%), with transgender women comprising 18.3% (37) of the sample (Table 2). A majority of participants were assigned a female sex at birth (154, 76.2%). Age distribution was 15–17 (66, 32.7%), 18–21 (94, 46.5%), or 22–24 years (42, 20.8%). Most identified as queer or pansexual (95, 47.0%), white (non-Hispanic; 135, 66.8%), were employed and/or a student, (163, 80.7%), had a high school education or general educational development (143, 70.8%), and did not report ever having experienced homelessness (163, 80.7%). Among the four census-designated U.S. regions, the sample was distributed as follows: 70 (34.6%) from the south, 58 (28.7%) from the midwest, 43 (21.3%) from the west, and 31 (15.4%) from the northeast.

**Table 2.** Demographics of Enrolled Participants

Demographics	Recruitment platform						Fisher's exact test
	Total	Facebook	Instagram	Craigslist	Referral	Other^a^	
	*n*=202	*n*=69	*n*=22	*n*=35	*n*=57	*n*=19	
	% (*n*)	% (*n*)	% (*n*)	% (*n*)	% (*n*)	% (*n*)	*p*
Gender identity							0.061
Trans men	40.6 (82)	36.2 (25)	45.5 (10)	51.4 (18)	43.9 (25)	21.1 (4)	
Trans women	18.3 (37)	26.1 (18)	—	11.4 (4)	19.3 (11)	21.1 (4)	
Nonbinary^b^	41.1 (83)	37.7 (26)	54.5 (12)	37.1 (13)	36.8 (21)	57.8 (11)	
Sex assigned at birth							0.100
Male	23.8 (48)	27.5 (19)	9.1 (2)	37.1 (13)	17.5 (10)	21.1 (4)	
Female	76.2 (154)	72.5 (50)	90.9 (20)	62.9 (22)	82.5 (47)	78.9 (15)	
Sexual orientation							0.118
Homosexual/gay	14.4 (29)	17.4 (12)	9.1 (2)	17.1 (6)	7.0 (4)	26.3 (5)	
Bisexual	23.8 (48)	26.1 (18)	22.7 (5)	25.7 (9)	28.1 (16)	—	
Queer/pansexual	47.0 (95)	43.5 (30)	63.6 (14)	40.0 (14)	49.1 (28)	47.4 (9)	
Other^c^	14.8 (30)	13.0 (9)	4.5 (1)	17.1 (6)	15.8 (9)	26.3 (5)	
Race							**0.000**
White/caucasian^d^	66.8 (135)	73.9 (51)	95.5 (21)	22.9 (8)	73.7 (42)	68.4 (13)	
Non-white^e^	33.2 (67)	26.1 (18)	4.5 (1)	77.1 (27)	26.3 (15)	31.6 (6)	
Age (years)							0.674
15–17	32.7 (66)	37.7 (26)	40.9 (9)	28.6 (10)	26.3 (15)	31.6 (6)	
18–21	46.5 (94)	46.4 (32)	31.8 (7)	54.3 (19)	47.4 (27)	47.3 (9)	
22–24	20.8 (42)	15.9 (11)	27.3 (6)	17.1 (6)	26.3 (15)	21.1 (4)	
U.S. region							**0.036**
Northeast	15.3 (31)	10.1 (7)	36.4 (8)	17.1 (6)	14.0 (8)	10.5 (2)	
Midwest	28.7 (58)	27.5 (19)	22.7 (5)	20.0 (7)	29.8 (17)	52.6 (10)	
South	34.7 (70)	39.1 (27)	13.6 (3)	31.4 (11)	43.9 (25)	21.1 (4)	
West	21.3 (43)	23.2 (16)	27.3 (6)	31.4 (11)	12.3 (7)	15.8 (3)	
Has graduated high school^f^							0.748
Yes	70.8 (143)	72.5 (50)	59.1 (13)	68.6 (24)	73.7 (42)	73.7 (14)	
No	29.2 (59)	27.5 (19)	40.9 (9)	31.4 (11)	26.3 (15)	26.3 (5)	
Employment status							0.244
Employed and/or student	80.7 (163)	75.4 (52)	72.7 (16)	80.0 (28)	89.5 (51)	84.2 (16)	
Unemployed, not a student	19.3 (39)	24.6 (17)	27.3 (6)	20.0 (7)	10.5 (6)	15.8 (3)	
Ever been homeless							0.214
Yes	19.3 (39)	23.2 (16)	31.8 (7)	17.1 (6)	14.0 (8)	10.5 (2)	
No	80.7 (163)	76.8 (53)	68.2 (15)	82.9 (29)	86.0 (49)	89.5 (17)	
Medical gender affirmation							0.736
Yes	30.7 (62)	33.3 (23)	18.2 (4)	31.4 (11)	33.3 (19)	26.3 (5)	
No, but plan to	47.0 (95)	47.8 (33)	54.5 (12)	51.4 (18)	43.9 (25)	36.8 (7)	
No, and do not plan to	22.3 (45)	18.9 (13)	27.3 (6)	17.1 (6)	22.8 (13)	36.8 (7)	
Currently living as most affirming gender							0.786
Yes	87.1 (176)	89.9 (62)	90.9 (20)	82.9 (29)	84.2 (48)	89.5 (17)	
No	12.9 (26)	10.1 (7)	9.1 (2)	17.1 (6)	15.8 (9)	10.5 (2)	

Bold indicates statistically significant values (*p*<0.05).

^a^ Includes nine through Tumblr, five through Twitter, one through study staff, three through newsletters/listservs, and one through locally distributed palm cards.

^b^ Includes 50 genderqueer/gender nonconforming, 25 agender/genderfluid, 7 nonbinary, and 1 two-spirit male.

^c^ Includes 9 heterosexual/straight, 3 asexual, 2 demisexual, 1 polysexual, and 1 sexually fluid, and 14 questioning/unsure.

^d^ Non-Hispanic white.

^e^ Includes 16 Hispanic, 12 black, 7 Asian, 3 Middle Eastern, 2 Native American/Alaskan Native, and 27 mixed.

^f^ Includes general educational development.

Fisher's exact tests revealed statistically significant variance of race (*p*=0.000) and region (*p*=0.036) by platform (Table 2). Although some trends between platforms may be observed among other variables, these did not achieve significance. Participants enrolled from Facebook (69, 34.2%) share a similar demographic makeup to that of the overall sample.

Compared with the overall sample, a significantly higher percentage of those enrolled from Instagram (22, 10.9%) identified as white (non-Hispanic; 21, 95.5%; *p*=0.002), and were from the northeast (8, 36.4%; *p*=0.003). Conversely, a significantly higher percentage of those enrolled from Craigslist (35, 17.3%) identified as non-white (including Hispanic; 27, 77.1%; *p*=0.000), comprising 40.3% of the non-white TY in the overall sample.

Few individuals were enrolled from other (19, 9.4%) routes, including Twitter (5, 2.5%), Tumblr (9, 4.4%), or recruitment through local organizations (5, 2.5%). These small subsamples have highly varied demographic makeups, and skew heavily toward midwest residence (10, 52.6%; *p*=0.011).

The total direct cost for all paid advertising was $2493.75 across the 12-month recruitment period, with $2482.73 spent on Facebook and $11.02 spent on Instagram. All other advertisements were placed using free, registered accounts across the various platforms. Cost can be broken down as follows: $0.08 per click, $0.92 per consent, $1.83 per eligibility screener, $3.57 per eligible participant, and $12.35 per baseline survey. The largest cost differential is observed between eligible participants and baseline surveys, which may be attributed to insufficient motivation to return to the study website for account setup, high levels of fraudulent account creation, or some combination of these factors.

## Discussion

Facebook generated the largest portion of the Project Moxie sample, but was also the most costly. Of the social media websites used, Facebook is the most popular among U.S. adults aged 18–24 years with 80% usage compared with 71% on Instagram and <50% on all others.^8^ For those aged 13–17 years in the United States, Facebook is less popular (51%) but second only to Instagram (72%).^7^

The advertising interface on Facebook and Instagram allowed TY to be targeted based on age and interests independent of subscription (e.g., friend request, follow), something unavailable through unpaid advertising routes. Although Facebook advertising took place throughout the entirety of recruitment, Instagram advertising only began approximately half way through this period. Although fewer individuals were recruited from Instagram than from Facebook, enrollment was similarly efficient.

Peer referral has been demonstrated as an effective recruitment strategy for hard-to-reach populations, including TY, the success of which relies on participating community members' connection to other interested individuals.^16,17^ In addition, previous data have shown that web-based referral to be successful in the recruitment of a diverse target population online.^18^ Data from Project Moxie support existing findings on the utility of participant referral, and demonstrate its viability in the efficient recruitment of TY online.

Placing free Craigslist advertisements was also a viable strategy for recruiting TY, especially those of color, with the highest enrollment rate in Project Moxie. Craigslist has been identified as an online space utilized by racially diverse SGM communities, with previous literature demonstrating the ability to recruit SGM samples of significantly lower Caucasian-identifying proportion through Craigslist.^19,20^ Although Craigslist provided a valuable opportunity to reach TY of color, it has limitations for use in research recruitment. Routine removal of research advertisements in some areas forced study staff to spend considerable time (4–6 hours per week) reposting advertisements on Craigslist.

Few individuals were enrolled using free post-based advertisements on either Twitter or Tumblr. Although Tumblr contributed the third highest volume of eligible individuals, it had an enrollment rate >20% lower than any other platform. Whether this is due to high levels of disinterest in participating or fraudulence is unknown. The ability to build and maintain a following, and/or network of reliable community members to share (e.g., retweet, reblog) advertisement posts to their own audience, is central to generating substantial exposure on these platforms without utilizing platform-provided advertising services. Future research should explore the establishment of effective community engagement protocols or use of paid advertisements when recruiting TY on these platforms.

### Limitations

Fraud is clearly a risk when recruiting online, making mechanisms of detection and mitigation essential in ensuring the validity of the enrolled sample. Although more than half of all Moxie accounts created were deemed fraudulent or duplicate, the implementation of protocol described in this report's methods allowed for verification of legitimate accounts and removal of fraudulent accounts. We did not collect data on the recruitment route for fraudulent participants, and thus it is not possible to determine whether differential fraudulence occurred between social media platforms.

Implementation of fraud detection mechanisms such as browser cookies or captcha during the registration process, in addition to retroactive fraud detection and mitigation (as used in Project Moxie), has the potential to save valuable resources and protect the integrity of online data.^21,22^

## Conclusion

Evidence from Project Moxie serves to add TY to the list of hard-to-reach populations who can be successfully recruited online.^3–6^ However, when recruiting online, consideration for both proactive and retroactive fraud detection and mitigation mechanisms is crucial to most effectively preserve the integrity of data.

## Abbreviations Used

IRB: Institutional Review Board
MI/CTR: motivational interviewing/counseling, testing, and referral
SGM: sexual and gender minority
TY: transgender youth
